# The role of haematological parameters in patients with COVID-19 and influenza virus infection

**DOI:** 10.1017/S095026882000271X

**Published:** 2020-11-05

**Authors:** Sumeyye Kazancioglu, Aliye Bastug, Bahadir Orkun Ozbay, Nizamettin Kemirtlek, Hurrem Bodur

**Affiliations:** 1 Department of Infectious Diseases and Clinical Microbiology, Ministry of Health Ankara City Hospital, Ankara, Turkey; 2 Department of Infectious Diseases and Clinical Microbiology, Ankara City Hospital, Health Science University Turkey, Ankara, Turkey

**Keywords:** COVID-19, delta neutrophil index, influenza, neutrophil-to-lymphocyte ratio, platelet-to-lymphocyte ratio, red blood cell

## Abstract

SARS-CoV-2, the causative agent of coronavirus disease 19 (COVID-19), was identified in Wuhan, China. Since then, the novel coronavirus started to be compared to influenza. The haematological parameters and inflammatory indexes are associated with severe illness in COVID-19 patients. In this study, the laboratory data of 120 COVID-19 patients, 100 influenza patients and 61 healthy controls were evaluated. Lower lymphocytes, eosinophils, basophils, platelets and higher delta neutrophil index (DNI), neutrophil-to-lymphocyte ratio (NLR) and platelet-to-lymphocyte ratio (PLR) were found in COVID-19 and influenza groups compared to healthy controls. The eosinophils, lymphocytes and PLR made the highest contribution to differentiate COVID-19 patients from healthy controls (area under the curves (AUCs): 0.819, 0.817 and 0.716, respectively; *P*-value is <0.0001 for all). The NLR, the optimal cut-off value was 3.58, which resulted in a sensitivity of 30.8 and a specificity of 100 (AUC: 0.677, *P* < 0.0001). Higher leucocytes, neutrophils, DNI, NLR, PLR and lower lymphocytes, red blood cells, haemoglobin, haematocrit levels were found in severe patients at the end of treatment. Nonsevere patients showed an upward trend for lymphocytes, eosinophils and platelets, and a downward trend for neutrophils, DNI, NLR and PLR. However, there was an increasing trend for eosinophils, platelets and PLR in severe patients. In conclusion, NLR and PLR can be used as biomarkers to distinguish COVID-19 patients from healthy people and to predict the severity of COVID-19. The increasing value of PLR during follow-up may be more useful compared to NLR to predict the disease severity.

## Introduction

In January 2020, the severe acute respiratory syndrome virus 2 (SARS-CoV-2) was identified in China and the disease was termed coronavirus disease 2019 (COVID-19) [1, 2]. Since then, this novel RNA beta-coronavirus began to be compared to the influenza virus. Both viruses that cause respiratory disease are transmitted by contact and droplets. As a result, the same public health measures, such as hand hygiene and good respiratory etiquette are important precautions to prevent infection. It has been observed that patients with COVID-19 and influenza could experience a range of clinical manifestations, from no symptoms to severe illness [3]. Recently, it has been reported that haematological parameters and inflammatory indexes based on blood cell analysis had an important predictive value for the prognosis of infections, and many other diseases [4–6]. To date, clinical and laboratory features such as lymphopoenia, elevated C-reactive protein (CRP), D-dimer and liver enzymes have been associated with severe COVID-19 [7, 8]. Moreover, haematological parameters and indexes such as the neutrophil-to-lymphocyte ratio (NLR) and platelet-to-lymphocyte ratio (PLR) were investigated as potential indicators of the severity of the COVID-19 [9, 10].

First, this study aimed to use haematological parameters (e.g. neutrophils, lymphocytes and platelets) and blood cell count indexes, particularly NLR, PLR and delta neutrophil index (DNI) to differentiate COVID-19 patients from influenza virus infection and healthy controls. Second, we analysed the alterations in laboratory parameters of 120 patients with COVID-19 to determine the predictors of severe illness.

## Materials and methods

### Study design and participants

The current study retrospectively enrolled 120 confirmed COVID-19 patients who were hospitalised in a tertiary hospital from 15 March to 30 April 2020. The diagnosis was confirmed by detecting SARS-CoV-2 RNA in oro-nasopharyngeal swab samples. A total of 100 patients were diagnosed with definite influenza infection by positive nucleic acid detection in throat swab samples of which 37 had influenza A and 24 had influenza B. The influenza group was chosen in the Northern Hemisphere influenza season from 1 October 2018 to 1 March 2019. Also, 61 healthy controls without any chronic disease and respiratory symptoms were recruited for the control group.

Demographic data and laboratory values were extracted from electronic medical records and patients' files. The following variables were recorded for each COVID-19 patient: age, sex, severity assessment on admission, laboratory findings of admission and end of treatment (the fifth day of hospitalisation). A complete blood count (CBC) was performed using the ADVIA 2120 Hematology System (Siemens Healthcare Diagnostics, Erlangen, Germany). Biochemical parameters were measured using Atellica Solution Immunassay & Clinical Chemistry Analyzers (Siemens Healthcare Diagnostics, Erlangen, Germany). Prothrombin time (PT), activated partial thromboplastin time, international normalised ratio (INR) and D-dimer were analysed using the Sysmex CS-5100 System (Siemens Healthcare Diagnostics, Erlangen, Germany). On admission, patients with COVID-19 were categorised into two groups (nonsevere and severe illness) according to the National Institutes of Health (NIH) classification based on disease severity [11].

The severe illness was defined as:

Respiratory frequency >30 breaths per min, SpO_2_ <94% on room air at sea level, a ratio of the arterial partial pressure of oxygen to fraction of inspired oxygen (PaO_2_/FiO_2_) <300 or lung infiltrates >50%.

Approval from the local ethics committee was obtained for this study (confirmation date and number: 21.05.2020/E1-20-617). This study was conducted by the principles of the Declaration of Helsinki.

### Statistical analysis

Statistical analyses were performed using SPSS software version 24.0. Comparisons for categorical variables were executed using the Pearson's chi-square test or Fisher's exact test. Kolmogorov–Smirnov test was performed to check the normality of the continuous variables. Differences between the two groups were compared using the Mann–Whitney *U* test. Kruskal–Wallis test was used for comparisons of more than two groups and the significant (*P* < 0.05) results from the Mann–Whitney test (with post-hoc Bonferroni correction) were analysed. The receiver operation characteristic (ROC) curve analysis was performed to determine the efficacy of various parameters in distinguishing the patients with COVID-19 from influenza and healthy controls. The ROC curve analysis was also used to predict the severity of COVID-19. Statistical significance was defined as *P* < 0.05.

## Results

### Laboratory parameters in COVID-19 and influenza patients and healthy controls on admission

The COVID-19 group median age was higher than the influenza group and healthy controls. There were several significant differences, including lower lymphocytes, eosinophils, basophils and platelets, and higher DNI, NLR and PLR were found in COVID-19 and influenza groups compared to healthy controls. The COVID-19 group had lower white blood cell (WBC) levels compared to healthy controls. The influenza group had higher neutrophils compared to the COVID-19 group. The influenza group had significantly lower red blood cells (RBCs), haemoglobin and haematocrit levels compared to the COVID-19 group and healthy controls and higher red cell distribution width (RDW) compared to healthy controls. The biochemical parameters, higher alanine aminotransferase (ALT), aspartate aminotransferase (AST) and lactate dehydrogenase (LDH) levels were found in the COVID-19 group compared to the influenza group and healthy controls. The increase of CRP was observed in COVID-19 and influenza groups compared to healthy controls (Table 1).

**Table 1.** Baseline laboratory parameters of patients with influenza, COVID-19 and healthy controls

	Normal range (unit)	Healthy controls (*n* = 61)	Influenza group (*n* = 100)	COVID-19 group (*n* = 120)	*χ*^2^	*P*
Age	–	28 (24–56)	28 (16–66)	45 (16–83)	78.264	***<0.001***
Male gender	–	34 (55.7%)	33 (33%)	72 (60%)	17.133	***<0.001***
Leucocytes	4200–10 800 (/μl)	6830 (3940–11 660)	5695 (2500–15 370)	5370 (2230–16 520)	16.222	***<0.001***
Neutrophils	1700–7900 (/μl)	4090 (1950–7530)	4385 (1150–12 960)	3365 (1110–13 770)	9.699	***0.008***
Lymphocytes	1500–4500 (/μl)	1960 (1020–3920)	795 (160–2750)	1265 (390–3030)	104.539	***<0.001***
Monocytes	100–900 (/μl)	380 (190–780)	425 (110–1240)	380 (40–1380)	5.077	0.079
Eosinophils	20–550 (/μl)	110 (30–620)	40 (0–440)	40 (0–290)	61.572	***<0.001***
Basophils	0–200 (/μl)	40 (10–200)	30 (0–330)	20 (0–600)	13.223	***0.001***
RBCs	4.3–5.75 (10^12^/l)	5.06 (3.79–7.02)	4.54 (2.97–5.79)	4.87 (3.97–5.82)	22.988	***<0.001***
Haemoglobin	13–16.6 (g/dl)	15 (10–17.3)	13.15 (7.4–17.4)	14.3 (9.6–17.7)	26.389	***<0.001***
Haematocrit	38–49 (%)	43.7 (31–49.6)	39 (26.3–48.9)	41.1 (15.2–52.5)	24.021	***<0.001***
RDW	11.5–16 (%)	13.1 (12–15.9)	13.6 (11.9–19.8)	13.4 (11.7–27.3)	6.404	***0.041***
Platelets	16 0000–383 000 (/μl)	254 000 (166 000–602 000)	227 000 (47 000–489 000)	219 500 (119 000–506 000)	14.097	***0.001***
Platecrit	0.12–0.36 (%)	0.21 (0.14–0.46)	0.18 (0.04–0.33)	0.18 (0.1–0.41)	18.934	***<0.001***
DNI	(%)	0.1 (0.1–0.82)	0.1 (0.1–12.1)	0.1 (0.1–9.5)	30.663	***<0.001***
NLR	–	1.89 (0.82–3.59)	5.72 (0.98–54)	2.61 (0.47–15.54)	64.398	***<0.001***
PLR	–	130 (60–230)	290 (70–1050)	180 (60–620)	82.671	***<0.001***
ALT	<50 (U/l)	20.5 (10–85)	21 (5–71)	25 (3–199)	17.766	***<0.001***
AST	<35 (U/l)	20 (10–41)	23 (1–61)	24 (1–188)	7.399	***0.025***
LDH	120–246 (U/l)	181 (135–361)	193 (42–327)	217.5 (123–697)	31.081	***<0.001***
CK	32–294 (U/l)	95 (49–361)	87 (32–1090)	104 (26–2183)	0.244	0.885
CRP	0–5 (g/l)	0.9 (0.1–24)	14.5 (3–299)	8 (0.6–198)	59.394	***<0.001***

RDW, red cell distribution width; MPV, mean platelet volume; DNI, delta neutrophil index; NLR, neutrophil-to-lymphocyte ratio; PLR, platelet-to-lymphocyte ratio; ALT, alanine aminotransferase; AST, aspartate aminotransferase; LDH, lactate dehydrogenase; RBCs, red blood cells; CRP, C-reactive protein; CK, creatinine kinase.

Pearson's *χ*^2^, Kruskal–Wallis *H* analysis. Data are *n* (%) or median (min-max).

The ROC curve analysis was performed to distinguish the patients with COVID-19 from healthy controls. Eosinophils, lymphocytes and PLR had the highest area under the curves (AUCs) in the ROC analysis (0.819, 0.817 and 0.716, respectively; *P*-value is <0.0001 for all). The NLR, the optimal cut-off value was 3.58, which resulted in a sensitivity of 30.8 and a specificity of 100 (AUC: 0.677, *P* < 0.0001).

To distinguish the patients with COVID-19 from influenza, the ROC curve was used. PLR, NLR and lymphocytes had the highest AUCs in the ROC analysis (0.746, 0.730 and 0.729, respectively; *P*-value is <0.0001 for all).

### Laboratory parameters on admission and end of treatment day with COVID-19 severity

On admission, patients with COVID-19 were categorised as nonsevere (*n* = 85) and severe (*n* = 35) groups. The severe group had an older median age compared to the nonsevere group (*P* < 0.001). The optimal cut-off value of age was found to be >48 when the ROC curve analysis was used (*P* < 0.0001, AUC: 0.82, sensitivity: 85.71, specificity: 71.76). The presence of hypertension, coronary artery disease and chronic obstructive pulmonary disease (COPD) was more common in the severe group significantly (*P* = 0.003, 0.022, 0.036, respectively). Higher leucocytes, neutrophils, AST, LDH, creatinine kinase (CK), PT, INR, D-dimer, CRP, interleukin-6 (IL-6), ferritin, NLR and PLR, and lower lymphocyte levels were found in the severe group on admission (Tables 2 and 3).

**Table 2.** Demographics and laboratory findings of patients with COVID-19

	Total (*n* = 120)	Nonsevere group (*n* = 85)	Severe group (*n* = 35)	*χ*^2^/*Z*	*P*
Age	45 (16–83)	39 (16–75)	59 (37–83)	−5.591	***<0.001***
Male gender	72 (60)	52 (61.2)	20 (57.1)	0.168	0.682
Diabetes	12 (10)	6 (7.1)	6 (17.1)	2.801	0.106
Hypertension	26 (21.7)	12 (14.1)	14 (40)	9.785	***0.003***
CAD	7 (5.8)	2 (2.4)	5 (14.3)	6.426	***0.022***
COPD	16 (13.3)	7 (8.2)	8 (22.9)	4.846	***0.036***
ALT	25 (3–199)	23 (3–199)	34 (10–112)	−1.958	0.05
AST	24 (1–188)	21 (1–188)	36 (11–85)	−4.896	***<0.001***
LDH	217.5 (123–697)	202 (123–506)	320 (185–697)	−6.386	***<0.001***
CK	104 (26–2183)	93 (26–1102)	140 (28–2183)	−2.446	***0.014***
PT	12.1 (10.5–18.7)	12 (10.5–14.4)	12.7 (10.7–18.7)	−2.842	***0.004***
APTT^a^	25.1 (5.9–43.2)	25.3 (5.9–43.2)	24.75 (19–31.4)	−1.116	0.265
INR^b^	1.02 (0.86–1.62)	1.02 (0.86–1.23)	1.08 (0.91–1.62)	−3.014	***0.003***
D-dimer^c^	0.4 (0.05–28.49)	0.34 (0.05–2.93)	0.65 (0.22–28.49)	−4.653	***<0.001***
CRP	8 (0.6–198)	5 (0.7–153)	55 (0.6–198)	−5.809	***<0.001***
IL-6^d^	21.2 (2–118)	3 (2–19)	28.6 (2.69–118)	−3.416	***0.001***
Ferritin^e^	127.5 (5.2–1580)	84.9 (5.2–823)	330 (30.8–1580)	−5.498	***<0.001***

ALT, alanine aminotransferase; AST, aspartate aminotransferase; LDH, lactate dehydrogenase; APTT, activated partial thromboplastin time; INR, international normalised ratio; CRP, C-reactive protein; PT, prothrombin time; CK, creatinine kinase; IL-6, interleukin-6.

Pearson's *χ*^2^, Fisher's exact test, Mann–Whitney *U* analysis. Data are *n* (%) or median (min-max).

a Normal range: 21–32 sn.

b Normal range: 0.8–1.2.

c Normal range: <0.55 mg/l.

d Normal range: 0–3.4 pg/dl.

e Normal range: 10–291 μg/l.

**Table 3.** Admission and end of treatment laboratory parameters of patients with COVID-19

	Admission				End of Treatment			
	Nonsevere group (*n* = 85)	Severe group (*n* = 35)	*Z*	*P*	Nonsevere Group (*n* = 85)	Severe Group (*n* = 35)	*Z*	*P*
Leucocytes	5150 (2230–10 850)	5970 (2810–16 520)	−1.986	***0.047***	5410 (662–11 770)	6710 (3460–14 340)	−3.456	***0.001***
Neutrophils	3110 (1110–8120)	4270 (1740–13 770)	−3.199	***0.001***	2830 (4040–7090)	4390 (3370–13 270)	−4.570	***<0.001***
Lymphocytes	1330 (390–3030)	1090 (560–2070)	−3.057	***0.002***	1770 (390–3800)	1080 (240–2690)	−4.885	***<0.001***
Monocytes	380 (40–940)	330 (40–1380)	−1.58	0.114	350 (140–890)	400 (190–770)	−1.395	0.163
Eosinophils	40 (0–290)	30 (0–150)	−1.637	0.102	80 (0–320)	90 (10–310)	−0.477	0.633
Basophils	20 (0–170)	30 (0–600)	−1.239	0.215	20 (0–160)	20 (10–70)	−1.746	0.081
RBCs	4.93 (3.98–5.82)	4.81 (3.97–5.79)	−1.62	0.105	4.72 (3.88–5.89)	4.57 (3.68–5.83)	−2.523	***0.012***
Haemoglobin	14.5 (9.6–17.7)	13.8 (11.2–17.4)	−1.733	0.083	13.8 (9.2–17.9)	12.6 (10.1–15.7)	−3.292	***0.001***
Haematocrit	42 (15.2–52.5)	40.2 (33.9–49.7)	−1.952	0.051	40.5 (29.8–53.5)	37.7 (31.1–96.6)	−2.339	***0.019***
RDW	13.3 (11.7–27.3)	13.5 (12.3–17.1)	−1.497	0.135	13.3 (11.9–17.6)	13.4 (12.1–17.2)	−1.381	0.167
Platelets	217000 (119000–488000)	221000 (131000–506000)	−0.110	0.913	231000 (104000–441000)	357000 (135000–654000)	−5.280	***<0.001***
Platecrit	0.18 (0.1–0.37)	0.18 (0.1–0.41)	−0.287	0.774	0.19 (0.08–0.37)	0.28 (0.12–0.57)	−5.283	***<0.001***
DNI	0.1 (0.1–9.5)	0.1 (0.1–4.4)	−1.794	0.073	0.1 (0.1–5.5)	0.1 (0–6.4)	−2.211	***0.027***
NLR	2.31 (0.47–9.63)	4.04 (1.51–15.54)	−4.027	***<0.001***	1.52 (0.57–7.29)	3.72 (0.64–40)	−6.12	***<0.001***
PLR	170 (60–620)	220 (100–490)	−2.792	***0.005***	130 (60–420)	300 (300–940)	−5.782	***<0.001***

CAD, coronary artery disease; COPD, chronic obstructive pulmonary disease; RDW, red cell distribution width; DNI, delta neutrophil index; NLR, neutrophil-to-lymphocyte ratio; PLR, platelet-to-lymphocyte ratio; RBCs, red blood cells.

Mann–Whitney *U* analysis. Data are median (min-max).

Higher leucocytes, neutrophils, DNI, NLR, PLR and platelets, and lower lymphocytes, RBCs, haemoglobin and haematocrit levels were found in the severe group at the end of treatment (Table 3).

In comparison of the admission and end of treatment laboratory values for each group, a significant increase of lymphocytes, eosinophils and platelets, and a decrease of neutrophils, DNI, NLR and PLR were found in the nonsevere group. In the severe group, a significant increase in eosinophils, platelets and PLR was found. A downward trend in RBC, haemoglobin and haematocrit levels was found in nonsevere and severe groups (Table 4).

**Table 4.** Evaluation of the admission and end of treatment laboratory parameters in nonsevere and severe groups with COVID-19

	Nonsevere group (*n* = 85)				Severe group (*n* = 35)			
	Admission	End of treatment	*Z*	*P*	Admission	End of treatment	*Z*	*P*
Leucocytes	5150 (2230–10 850)	5410 (662–11 770)	−0.082	0.935	5970 (2810–16 520)	6710 (3460–14 340)	−1.245	0.213
Neutrophils	3110 (1110–8120)	2830 (4040–7090)	−2.498	***0.013***	4270 (1740–13 770)	4390 (3370–13 270)	−0.082	0.935
Lymphocytes	1330 (390–3030)	1770 (390–3800)	−5.258	***<0.001***	1090 (560–2070)	1080 (240–2690)	−1.786	0.074
Monocytes	380 (40–940)	350 (140–890)	−0.875	0.382	330 (40–1380)	400 (190–770)	−1.753	0.08
Eosinophils	40 (0–290)	80 (0–320)	−4.391	***<0.001***	30 (0–150)	90 (10–310)	−3.739	<***0.001***
Basophils	20 (0–170)	20 (0–160)	−1.251	0.211	30 (0–600)	20 (10–70)	−1.003	0.316
RBCs	4.93 (3.98–5.82)	4.72 (3.88–5.89)	−4.307	***<0.001***	4.81 (3.97–5.79)	4.57 (3.68–5.83)	−3.686	***<0.001***
Haemoglobin	14.5 (9.6–17.7)	13.8 (9.2–17.9)	−4.617	***<0.001***	13.8 (11.2–17.4)	12.6 (10.1–15.7)	−4.359	***<0.001***
Haematocrit	42 (15.2–52.5)	40.5 (29.8–53.5)	−3.874	***<0.001***	40.2 (33.9–49.7)	37.7 (31.1–96.6)	−2.933	***0.003***
RDW	13.3 (11.7–27.3)	13.3 (11.9–17.6)	−1.855	0.064	13.5 (12.3–17.1)	13.4 (12.1–17.2)	−1.521	0.128
Platelets	217000 (119000–488000)	231000 (104000–441000)	−2.268	***0.023***	221000 (131000–506000)	357000 (135000–654000)	−4.66	***<0.001***
Platecrit	0.18 (0.1–0.37)	0.19 (0.08–0.37)	−2.123	***0.034***	0.18 (0.1–0.41)	0.28 (0.12–0.57)	−4.588	***<0.001***
DNI	0.1 (0.1–9.5)	0.1 (0.1–5.5)	−2.769	***0.006***	0.1 (0.1–4.4)	0.1 (0–6.4)	−0.056	0.955
NLR	2.31 (0.47–9.63)	1.52 (0.57–7.29)	−5.335	***<0.001***	4.04 (1.51–15.54)	3.72 (0.64–40)	−0.901	0.368
PLR	170 (60–620)	130 (60–420)	−4.237	***<0.001***	220 (100–490)	300 (300–940)	−3.317	***0.001***

RDW, red cell distribution width; MPV, mean platelet volume; PDW, platelet distribution width; DNI, delta neutrophil index; NLR, neutrophil-to-lymphocyte ratio; PLR, platelet-to-lymphocyte ratio; RBCs, red blood cells.

Wilcoxon's signed ranks analysis. Data are median (min-max).

## Discussion

Since the SARS-CoV-2 was identified, this novel virus began to be compared to influenza. First, we described laboratory parameters in COVID-19 and influenza patients with healthy controls. Also, the admission and end of treatment (the fifth day of hospitalisation) values of these laboratory parameters were investigated in COVID-19 patients with disease severity.

In recent years, several biomarkers of systemic inflammation have become available as part of the expanded CBC. These biomarkers based on CBC are investigated in several areas because they are simple and low cost, so many clinicians can easily use it in practice. According to the studies conducted with COVID-19 the haematology laboratory plays an important role in providing several useful prognostic markers [12]. In the current study, lower lymphocytes, eosinophils, basophils and platelets, and higher DNI, NLR and PLR were found in COVID-19 and influenza groups compared to healthy controls. Lymphopoenia has been a common finding in influenza infection [6]. Lymphopoenia has also been a common finding in patients with COVID-19 [13]. Eosinophils which are a small part of leucocytes have been shown to have various other functions, including immunoregulation and antiviral activity. It has been reported in studies that eosinopoenia was detected in COVID-19 patients and may help predict severe prognosis [14, 15]. NLR and PLR were found to be useful indicators for diagnosis and differentiation of influenza A infection [16]. Yang *et al*. investigated the diagnostic and predictive role of NLR and PLR in COVID-19 patients, and they found these indexes were useful [10].

Our findings showed that parameters including eosinophils, lymphocytes and PLR made the highest contribution to differentiate the COVID-19 patients from healthy controls. Similarly, Sun *et al*. showed lower eosinophils and lymphocytes, and a higher PLR in patients with COVID-19 compared to controls [17]. The lymphocytes, NLR and PLR values were seen as more useful than other parameters to distinguish patients with COVID-19 from influenza according to this study.

The DNI, which is a calculated parameter that reflects the ratio of immature granulocytes over total neutrophil count in the peripheral blood, was previously reported to be more predictive of infection and prognosis than WBC counts and CRP [18]. In this study, the increase of DNI was observed in both COVID-19 and influenza groups compared to healthy controls. The DNI may be a useful parameter for viral respiratory infections compared to other various leucocyte-related parameters such as the total WBC and neutrophil counts.

Currently, it is well known that influenza viruses can agglutinate erythrocytes by binding to sialic acid receptors on the host cell [19]. Our data demonstrated that the influenza group had lower RBC, haemoglobin and haematocrit levels compared to the COVID-19 group and healthy controls. Higher RDW was found in influenza patients compared to healthy controls, although there was no difference between the COVID-19 group and the other groups. Topaz *et al*. showed that higher RDW was a predictor of severe hospital complications in patients with influenza [20]. Foy *et al*. showed a relation between elevated RDW (>14.5%) and mortality in COVID-19 patients [21]. One centre study showed that there was no difference in RBC and haemoglobin levels in COVID-19 patients compared to controls [22]. However, it is not yet fully known whether SARS-CoV-2 affects erythrocytes.

The biochemical parameters, higher ALT, AST and LDH levels were found in the COVID-19 group compared to the influenza group and healthy controls. This situation can be explained by pneumonia and severe infection would be higher in COVID-19 compared to what is observed for influenza infection. The increase of CRP was observed in COVID-19 and influenza groups compared to healthy controls. CRP is used clinically as a biomarker for various inflammatory conditions; a rise in CRP levels is determined in both COVID-19 and influenza groups.

Older age, hypertension, coronary artery disease and COPD were reported in various studies as predictors for severe COVID-19 similar to this study [23–26]. In this study, higher leucocytes, neutrophils, AST, LDH, CK, PT, INR, D-dimer, CRP, IL-6, ferritin, NLR and PLR, and lower lymphocyte levels were found in the severe group on admission. The comprehensive review by Kermali *et al*. mentioned that the increase of NLR, CRP, LDH and IL-6, and the decrease of lymphocyte was associated with severe COVID-19 [8]. Liu *et al*. showed that on admission, the levels of IL-6, CRP, LDH and ferritin were closely related to the severity of COVID-19. Virus-infected cells lead to cytokine storm and this is observed as an increase in IL-6 levels in COVID-19 [27, 28]. A study showed that when LDH and CRP levels were correlated with computed tomography scans, significantly higher levels reflected the severity of pneumonia [29]. Our analysis also revealed that NLR and PLR might be used to evaluate severe patients with COVID-19. The optimal cut-off values for PLR and NLR were 230 (*P* < 0.0001, AUC: 0.734, sensitivity: 77.14, specificity: 58.82) and 2.47 (*P* = 0.005, AUC: 0.663, sensitivity: 45.71, specificity: 89.41), respectively, in this study. Yang *et al*. found that the optimal cut-off values were 3.3 and 180 for NLR and PLR, respectively. Also, they highlighted that NLR and age are recommended as practical tools to evaluate the severity of COVID-19 patients [10].

Significantly higher leucocytes, neutrophils, DNI, NLR, PLR and platelets, and lower lymphocytes, RBC, haemoglobin and haematocrit levels were found in the severe group at the end of treatment. With these results, it can be said that the impairment in haematological parameters is related to the severity of COVID-19. When the difference in the laboratory values between admission and end of treatment was evaluated, a downward trend in RBC, haemoglobin and haematocrit levels was found in nonsevere and severe groups. The previous studies reported that lower haemoglobin levels were shown in patients with COVID-19 [17, 30]. These findings can suggest that SARS-CoV-2 may affect the RBC system in accordance with the previous studies [17]. Nonsevere patients showed an upward trend for lymphocytes, eosinophils and platelets, and a downward trend for neutrophils. However, severe patients had an increasing trend with eosinophils, platelets and PLR. Similarly, Liu *et al*. observed that 13 severe cases showed significant and sustained decreases in lymphocytes count but increases in neutrophil counts than 27 mild cases [28]. Chen *et al*. showed that restored levels of lymphocytes, eosinophils and platelets could serve as the predictors of recovery, whereas progressive increases in neutrophils, basophils and IL-6 were risk factors for fatal outcomes of COVID-19 [15]. These results suggest that an insufficient improvement in abnormal laboratory parameters may be a predictor for severe illness. In severe patients, although no change was observed in DNI and NLR at the end of treatment, the increase in PLR was detected. This situation can be interpreted as elevated PLR progression is more useful than NLR and DNI to predict the disease severity. No relation was found between eosinophil counts and the severity of the disease. On the other hand, the increase was shown in eosinophils between admission and end of treatment day in severe and nonsevere groups. A study that evaluated 10 COVID-19 patients showed that eosinophil values were low on admission, then all returned to normal before discharge [31].

In conclusion, respiratory infections are common and one of the leading causes of morbidity and mortality. Of respiratory infections, influenza is the most well studied viral infection and is commonly reported as the cause of epidemics. However, since the beginning of 2020, SARS-CoV-2 has become the most researched respiratory infection. The current study revealed that blood cell count analysis is a simple, cost-effective and rapid laboratory diagnostic basis for evaluating infectious inflammatory responses to respiratory infection. Also, the parameters based on CBC can be useful to predict the severity and the course of COVID-19.

## Data Availability

The authors confirm that the data supporting the findings of this study are available in the Supplementary materials.

## Supplementary material

For supplementary material accompanying this paper visit https://doi.org/10.1017/S095026882000271X. click here to view supplementary material
